# Effects of unexpected event urgency and flight scenario familiarity on pilot trainees performance and stress responses

**DOI:** 10.3389/fphys.2025.1599122

**Published:** 2025-07-14

**Authors:** Xing Peng, Qingfei Niu, Yaowei Liang, Yuchuan Luo, Ning Lu, Xiuyi Li

**Affiliations:** ^1^ School of Flight Technology, Civil Aviation Flight University of China, Guanghan, Sichuan, China; ^2^ Sichuan Provincial Engineering Research Center of Domestic Civil Aircraft Flight and Operation Support, Guanghan, Sichuan, China; ^3^ Department of Mechanical and Industrial Engineering, University of Toronto, Toronto, ON, Canada; ^4^ School of Art and Design, Xihua University, Chengdu, Sichuan, China; ^5^ CAAC Academy, Civil Aviation Flight University of China, Guanghan, Sichuan, China

**Keywords:** flight scenario, urgency, anxiety, stress response, pilot performance, flight simulation, familiarity

## Abstract

Pilot trainees’ ability to manage unexpected events is crucial for aviation safety, yet the impact of event urgency and flight scenario familiarity on pilot performance remains under-explored. This study investigates how different urgency levels of unexpected events influence pilot trainees’ flight performance, heart rate, and anxiety in both familiar (single-engine) and less familiar (twin-engine) flight scenarios. Two controlled experiments were conducted using flight simulators: Experiment 1 involved 27 pilot trainees operating a Cessna 172 single-engine simulator under low-urgency and high-urgency conditions, while Experiment 2 involved 25 pilot trainees using a FTD D40/D42 twin-engine simulator, introducing an additional no-event baseline. In the single-engine flight scenario, high-urgency unexpected events significantly impaired pilot trainees’ performance (Cohen’*d* = 0.454) and increased anxiety (η_p_^2^ = 0.229). In the twin-engine flight scenario, high-urgency unexpected events significantly impaired flight performance (MEI increased, η_p_^2^ = 0.737), elevated heart rate (η_p_^2^ = 0.516), and increased anxiety levels (η_p_^2^ = 0.442) compared to low-urgency events, which had minimal effects and, in some cases, improved pilot trainees focus. Additionally, pilot trainees performed better and exhibited lower anxiety in familiar scenarios, suggesting that task familiarity mitigates the negative impact of high-urgency unexpected events. These findings highlight the importance of incorporating urgency-based training scenarios and cross-aircraft training to enhance pilot trainees’ adaptive responses to unexpected events, ultimately improving flight safety.

## 1 Introduction

Given the extreme importance of pilots in ensuring flight safety, they must undergo rigorous training to enhance their flight skills (Muecklich et al., 2023; Obeng et al., 2024). The corresponding training has significantly improved flight safety, with the number of aviation accidents decreasing from 40 fatal accidents per million flights in 1959 to 0.14 per million flights in recent 5 years (International Air Transport Association, 2022). Nevertheless, accidents still occur because training cannot fully cover the dynamic situations and unexpected events pilots face when performing specific flight tasks. Unexpected events challenge pilots’ cognitive and operational capabilities, affecting their judgment and handling of malfunctions. These errors frequently occur in response to unexpected events, which challenge pilots’ cognitive and operational capacities, even after extensive simulator training. Reports from the U.S. National Transportation Safety Board (NTSB) highlight that unexpected events frequently occur during flights. For example, Colgan Air Flight 3407 crashed in Clarence Center, New York in 2009, was attributed to pilot misjudgment caused by unexpected stall—the crew incorrectly performed a stick-pull maneuver during a stall, overriding the automatic safety system and leading to a fatal crash (National Transportation Safety Board, 2010). Such events underline the necessity of clearly differentiating urgency levels of unexpected events to better understand pilots’ response mechanism.

In aviation, urgency levels of unexpected events are typically defined based on the immediacy of required action and potential impact on flight safety (Landy et al., 1991). High-urgency unexpected events necessitate immediate intervention due to the aircraft entering hazardous states or experiencing critical system failures, such as aerodynamic stalls, engine failure, or significant loss of cabin pressure. Conversely, low-urgency unexpected events include situations that require pilot attention but not immediate corrective actions, such as minor system failures or instrument malfunctions. Although these events may not immediately threaten flight safety, failure to address them effectively could escalate to serious consequences.

Previous studies have shown that unexpected events affect the performance, physiological, and psychological states, with measurable changes observed across multiple indicators (Little et al., 1999). In terms of performance, unexpected events have been shown to prolong reaction time, increase the probability of operational errors, and disrupt the execution of standard operating procedures, ultimately impairing task performance (Casner et al., 2013; Landman et al., 2020; Martin et al., 2015). Physiologically, unexpected events can induce elevated heart rate, increased galvanic skin response, and pupil dilation—all indicators of heightened emotional arousal (Agha, 2020; Kinney and O'Hare, 2020). Psychologically, unexpected events contribute to greater emotional volatility and negative affect, leading to higher self-reported anxiety and frustration (Agha, 2020; Eysenck et al., 2007; Landman et al., 2017; Ryffel et al., 2019).

Despite these general findings, the impact of unexpected events on pilots varies considerably across studies. For example, Kinney and O'Hare (2020) reported that pilots encountering unexpected engine failures exhibited significantly lower landing rate compared to those who anticipated such failures. In contrast, Casner et al. (2013) found no significant performance differences between expected and unexpected conditions (Casner et al., 2013; Kinney and O'Hare, 2020). These discrepancies suggest that additional moderating factors, such as flight scenario complexity and pilot familiarity, may influence how pilots respond to unexpected events.

Previous research indicates pilots perform more efficiently in familiar scenarios, as greater accumulated experience facilitates better management of unexpected events. For example, Agha (2020) found higher physiological stress and workload responses in multi-engine scenarios compared to single-engine scenarios under identical failures. These findings emphasize the importance of understanding how flight scenario familiarity interacts with event urgency to affect pilot performance (Agha, 2020).

However, how the urgency of unexpected events impact pilots’ physiological, psychological, and performance responses has rarely been systematically explored. Moreover, little is known about whether familiarity with the flight scenario. We design two simulation experiments to systematically investigate the urgency level of unexpected events and familiarity with flight scenarios: Experiment 1 (Single-engine scenario): pilot trainees operated a Cessna 172 flight simulator, where unexpected events were categorized into low-urgency events, referring to system failures requiring attention but not immediate action (e.g., instrument display failure), and high-urgency events, referring to critical malfunctions requiring immediate corrective action (e.g., aerodynamic stall) (Diarra et al., 2023; Rivera et al., 2014). Experiment 2 (Twin-engine scenario): pilot trainees operated a DA-42 flight simulator, incorporating an additional no-event baseline condition. The high-urgency event was modified to engine failure, reflecting a more complex and demanding operational environment. Based on the above content, we propose the following hypotheses:

Hypothesis 1a Compared to low-urgency unexpected events, high-urgency unexpected events will lead to significantly worse performance.

Hypothesis 1b Compared to low-urgency unexpected events, high-urgency unexpected events will lead to significantly higher heart rate.

Hypothesis 1c Compared to low-urgency unexpected events, high-urgency unexpected events will lead to significantly higher state anxiety scores.

Hypothesis 2 How might flight scenario familiarity (single-engine vs. twin-engine) influence the effect of unexpected event urgency on pilot trainees’ performance, heart rate, and anxiety scores?

To test Hypothesis 1a, Hypothesis 1b, and Hypothesis 1c, Experiments 1 and 2 introduced unexpected events with low-urgency and high-urgency levels, collecting performance, physiological, and psychological indicators to examine outcome differences across urgency conditions. For Hypothesis H2, Experiment 2 manipulated flight scenario familiarity by replacing the single-engine scenario (Experiment 1) with a twin-engine scenario, examining its moderating effect on unexpected events urgency outcomes. By systematically assessing pilot trainees’ physiological, psychological, and performance responses across varying scenarios, this research aims to clarify how event urgency and flight scenario complexity interact. Ultimately, these insights have significant implications for improving pilot trainees training, operational decision-making, and aviation safety management.

## 2 Experiment 1 (single-engine scenario)

### 2.1 Method

To test Hypothesis 1a, Hypothesis 1b, and Hypothesis 1c, a one-way within-subjects design was implemented in the Experiment. The independent variable “urgency” was manipulated via unexpected events at two levels: low and high. Concomitantly, participants’ performance (MEI), physiological indicators (HR, HRV), and psychological indicators (STAI) were recorded.

#### 2.1.1 Participants

We used G*Power 3.1.9.7 to conduct a power test. With effect size (d) of 0.69 (Kraus et al., 2020), power (1–β) of 0.8, and α level of 0.05 used in the pairwise comparison (Faul et al., 2007; Kang, 2021). This calculation indicated that approximately nineteen participants were needed. Based on this, twenty-seven participants (27 males, 22–24 years, *M*_age_ = 23.33, *SD*_age_ = 0.62) were recruited from the Civil Aviation Flight University of China (CAFUC). All pilot trainees participants held commercial aviation licenses from the Civil Aviation Administration of China (CAAC) and had logged an average of over 230 h of flight time (231–295 h, *M*_hour_ = 240.67, *SD*_hour_ = 14.02) in simulators and real aircraft. All participants reported normal or corrected-to-normal vision and hearing, with no history of neuropsychiatric illnesses or Spatial Disorientation. Before the experiment, all participants provided written informed consent. All procedures were conducted in accordance with prescribed ethical standards, and the ethics committee of the Civil Aviation Flight University of China approved the protocols. This research complied with the 1964 Helsinki Declaration. Each participant received a $10 payment after completing the experiment.

#### 2.1.2 Materials

**FIGURE 1:**
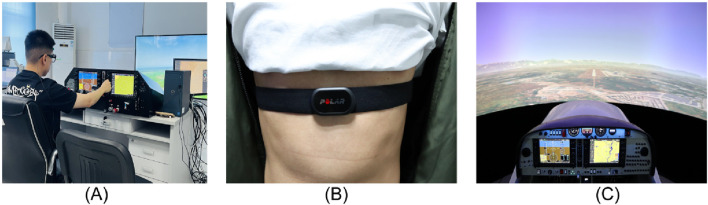
**(A)** is Experiment 1’s scenario based on the Cessna 172 single-engine simulator. **(B)** is the heart rate belt utilized in Experiment 1 and 2 is accompanied by a diagram illustrating its correct wearing. And **(C)** is Experiment 2’s scenario based on the FTD D40/D42 twin-engine simulator.

**FIGURE 2:**
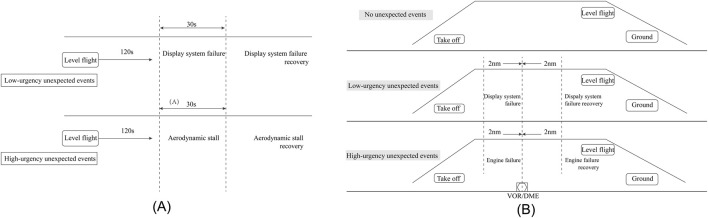
The flowchart of Experiment 1 **(A)** illustrates the differences in the start and end of the unexpected events and the settings for low and high urgency unexpected events. The flowchart of Experiment 2 **(B)** shows the flight process that participants have to complete when no unexpected events occur, the types of unexpected events that participants encounter, and the nodes at which the failure starts and ends in the low and high urgency conditions.

The experimental platform is based on a Cessna-172 light single-engine aircraft simulator, featuring simulated integrated avionics, force-feedback control architecture, and a high-fidelity aerodynamic model (Figure 1A). A POLAR heart rate belt (Figure 2B) is used for heart rate acquisition.

#### 2.1.3 Stimuli

To determine suitable unexpected events for different urgency levels, we strictly adhered to the operational guidelines in the Airdrop Manual. Subsequently, we conducted a structured Delphi consultation with 35 pilot instructors holding commercial pilot licenses and a mean flight experience of 4,252.66 h. Experts evaluated candidate events based on urgency, complexity, operational realism, and ecological validity. Following two rounds of consultation, an aerodynamic stall was selected as a representative high-urgency event for the single-engine scenario (Experiment 1), and engine failure was chosen for the more complex twin-engine scenario (Experiment 2) (Brinkman and Visser, 2007; Parnell et al., 2021). Low-urgency events in both scenarios involved minor instrumentation failures, consistently rated as requiring attention but no immediate corrective action. Additionally, Experiment 2 introduced the condition of no unexpected events, which aimed to provide a clearer understanding of the adverse effects of unexpected events on the normal mission.

#### 2.1.4 Procedures

The experiment required participants to complete two tasks, each lasting approximately 15 min. The order in which participants completed the two tasks was counterbalanced to minimize the impact of the learning effect.

The task containing the low-urgency unexpected event required the participant to maintain level flight at 90 knots, a heading of 200°, and an altitude of 5,000 ft. After 2 min, the attitude and heading instruments on the aircraft’s PFD failed. The participant was required to maintain level flight based on the alternate instrument located in the lower-right portion of the PFD and external visual references. Then, after 30 s, the attitude and heading instruments are recovered. The participant continued to maintain level flight. The task that included the high-urgency unexpected event required the experimental participant to maintain level flight at 90 knots, 100° heading, and 5,500 ft. After 2 min, a sudden increase in the tail-wind resulted in an aerodynamic stall of the aircraft. The participant is required to follow standard operating procedures to exit the stall and return to 90 knots, 100° heading, and 5,500 ft. After 30 s, the tailwind gradually decreased and the participants continued to maintain level flight (Figure 2A).

### 2.2 Data analysis

When measuring pilot trainees’ flight performance data during unexpected events, we calculated the Maneuver Error Index (MEI) using the method proposed by Alaimo et al. (Alaimo et al., 2020). It measures the relative difference between the flight data recorded by the simulator and the standard values. In this study, the flight data of heading (Equation 2), altitude (Equation 3), and speed (Equation 4) were selected to calculate the experimental participants’ MEI in these three aspects and summed to obtain the total MEI (Equation 1). Where $t_{j}$ and $t_{k}$ represent the selected period, $\psi_{i}\left(t\right)$ represents the heading as a function of time, and $\psi_{r}\left(t\right)$ represents the standard value of heading specified by the procedure. The synthesis considers in-flight data to give a more accurate representation of the performance levels of the participants.

$$MEI={MEI}_{HDG}+{\mathrm{MEI}}_{ALT}+{MEI}_{SPD}$$  (1)

$${MEI}_{HDG}=\frac{1}{\left(t_{j}-t_{k}\right)\cdot {\bar{\psi }}_{r}}\int_{t_{k}}^{t_{j}}\left|\psi_{i}\left(t\right)-\psi_{r}\left(t\right)\right|\;dt$$ (2)

$${MEI}_{ALT}=\frac{1}{\left(t_{j}-t_{k}\right)\cdot {\bar{z}}_{r}}\int_{t_{k}}^{t_{j}}\left|z_{i}\left(t\right)-z_{r}\left(t\right)\right|\;dt\;$$ (3)

$${MEI}_{SPD}=\frac{1}{\left(t_{j}-t_{k}\right)\cdot {\bar{\varphi }}_{r}}\int_{t_{k}}^{t_{j}}\left|\varphi_{i}\left(t\right)-\varphi_{r}\left(t\right)\right|\;dt$$ (4)

Heart rate indicators are used to characterize a pilot’s physiological state. The experiment collected participants’ heart rate (HR) and calculated their Heart Rate Variability (HRV), which refers to the small difference between heartbeat-to-heartbeat RR intervals. The sympathetic and vagus nerves coordinate with each other to maintain normal cardiac activity and normal heart rate variability. An imbalance in the coordination between the two will result in dysfunction of the cardiovascular system, leading to serious arrhythmia. Therefore, the time-domain characteristics of HRV in pilots can be used as an important index to reflect the function of autonomic nerves, cardiovascular regulation, and normal or abnormal cardiac activity. In our study, we selected the mean value of RR intervals (MEAN NNI), the Standard Deviation of RR intervals (SDNN), the Cardiac Vagal Index (CVI) and the Cardiac Sympathetic Index (CSI) as HRV indicators. Among these, MEAN NNI reflects individual stress levels (Kim et al., 2007), while SDNN assesses heart rate variability over a specific period (Hermida, 2003; Kim et al., 2018; Reyes Del Paso et al., 2013). CVI and CSI measure the regulatory capacity of the sympathetic and parasympathetic nerves on the heart, serving as physiological indicators of stress levels (Porges, 1995). They serve as direct indicators of overall HRV magnitude and variability, and are widely used for pilot stress monitoring (Diarra et al., 2023; Regula et al., 2014; Van Weelden et al., 2022).

The anxiety indicators are based on the State-Trait Anxiety Inventory (STAI), a self-rating scale characterized by its simplicity, validity, and ease of analysis, and is the definitive measure of anxiety in adults. The STAI clearly distinguishes between the transient states of “state anxiety” and the more general and long-lasting qualities of “trait anxiety.” Since the anxiety examined in our study focuses on pilot trainees’ state anxiety during unexpected events, the data collection specifically utilized the state anxiety portion of the scale to measure their anxiety scores.

### 2.3 Results

#### 2.3.1 Maneuver Error Index

**FIGURE 3:**
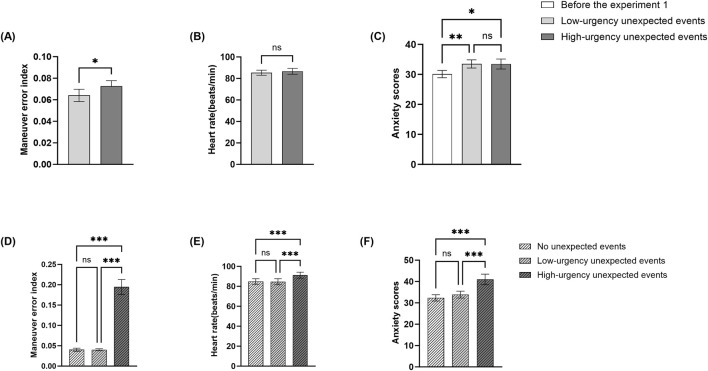
Maneuver Error Index **(A)**, Heart rate **(B)**, and Anxiety scores **(C)** results for different urgency conditions in the single-engine flight scenario. Maneuver Error Index **(D)**, Heart rate **(E)**, and Anxiety scores **(F)** results for different urgency conditions in the twin-engine flight scenario. Error bars represent ± *SE*, ns is *p* > 0.05, *p < 0.05, **p < 0.01, ***p < 0.001.

As shown in Figure 3A, a paired samples t-test is conducted on participants’ MEI. Results revealed that high-urgency unexpected events significantly impaired pilot trainees’ flight performance, with MEI under high-urgency unexpected events (*M* = 0.073, *SD* = 0.027) being statistically higher than those under low-urgency ones (*M* = 0.064, *SD* = 0.030) (*t* = −2.360, *p* = 0.026, Cohen’*d* = 0.454, 95%*CI* [−0.016, −0.001]). Hypothesis 1a was supported by this result.

Using MATLAB as the computational platform and running the TRAPZ code to generate Supplementary Figure S1 (see Supplementary Material), which illustrates the temporal variation in speed, altitude, and heading for a participant during the flight task (Landman et al., 2017; Landman et al., 2018; Musicant et al., 2018). The differences between the actual and targeted values are illustrated for a pilot trainee experiencing low and high levels of urgency in response to unexpected events, respectively, in the single-engine flight scenario. As illustrated by the example, the differences between the actual and targeted speed, altitude, and heading are more significant when the pilot trainee encounters a high-urgency unexpected event than a low-urgency unexpected event.

#### 2.3.2 Heart rate

As shown in Figure 3B, a paired-sample t-test is conducted on participants’ HR. The results revealed that pilot trainees exhibited higher HR during high-urgency unexpected events (*M* = 86.624, *SD* = 14.306) compared to low-urgency unexpected events (*M* = 85.317*, SD* = 12.344), though this difference is not significant [*t* = −1.933, *p* = 0.064, Cohen’*d* = 0.372, 95%*CI* (−2.696, 0.083)]. Hypothesis 1b was not supported.

#### 2.3.3 Heart rate variability

As shown in Table 1, paired-sample t-tests are conducted on participants’ MEAN NNI, SDNN, CSI and CVI measures. The results revealed that only CSI demonstrated a significant difference [*t* = −3.090, *p* = 0.005, Cohen’*d* = 0.595, 95%*CI* (−0.831, −0.167)], with higher CSI observed under high-urgency unexpected events (*M* = 4.24, *SD* = 2.01) compared to low-urgency conditions (*M* = 3.74, *SD* = 1.65). The remaining three HRV indicators showed no significant differences between the two urgency levels of unexpected events.

**TABLE 1:** Descriptive statistics of Heart Rate Variability results in the single-engine (Experiment 1) and the twin-engine (Experiment 2) flight scenario.

Experiment	Indicators	No	Low-urgency	High-urgency
Experiment 1	MEAN NNI	none	715.62 ± 97.10	709.58 ± 106.90
	SDNN	none	45.98 ± 15.10	43.09 ± 14.86
	CSI	none	3.74 ± 1.65	4.24 ± 2.01
	CVI	none	4.16 ± 0.38	4.14 ± 0.38
Experiment 2	MEAN NNI	730.38 ± 122.8	734.02 ± 128.47	684.60 ± 108.47
	SDNN	76.27 ± 35.17	52.52 ± 23.76	51.68 ± 18.89
	CSI	3.51 ± 1.19	3.31 ± 1.00	6.04 ± 3.37
	CVI	4.44 ± 0.40	4.32 ± 0.39	4.29 ± 0.44

*Note*. The data is made up of *M* ± *SD*., no is no unexpected events, Low-urgency is Low-urgency unexpected events and High-urgency is High-urgency unexpected events.

#### 2.3.4 Anxiety

Indicators of participants’ state anxiety are collected using STAI before the start of the experiment, after low-urgency unexpected event and high-urgency unexpected event respectively. Therefore, we conducted a one-way repeated measures ANOVA (Figure 3C) on anxiety levels. Results revealed a significant main effect of the type of unexpected events (*F*_(2,25)_ = 7.736, *p* = 0.003, η_p_^2^ = 0.229). Post hoc multiple comparisons showed pilot trainees’ pre-experiment anxiety levels (*M* = 30.11, *SD* = 6.31) were significantly lower than those during both low- (*M* = 33.48, *SD* = 7.18) and high-urgency unexpected events (*M* = 33.44, *SD* = 8.61). Hypothesis 1c was not supported.

The data analysis indicates that high-urgency unexpected events in single-engine flight scenarios had a more significant negative influence on pilot trainees performance than low-urgency unexpected events. However, unlike the MEI indicators, no significant differences were observed in heart rate and anxiety levels. One potential explanation for this difference is that the participants were more familiar with the Cessna 172 and had flown on this model for a longer time, and thus had some expectation of potential malfunctions occurring. Moreover, the single-engine flight scenarios are relatively simple in construction and system setup, and their operation is not as complicated as the twin-engine flight scenarios. Consequently, the participant’s competence to cope with unexpected events is less demanding, thus making it easier for them to cope with both malfunctions.

## 3 Experiment 2 (twin-engine scenario)

### 3.1 Method

To examine whether flight scenario familiarity moderates the effects of unexpected event urgency on pilot trainees’ performance, physiological, and psychological responses, we manipulated flight scenarios—switching from the familiar single-engine scenario to the less familiar twin-engine scenario. A one-way within-subjects design was implemented in this Experiment. The independent variable 'urgency’ was manipulated via unexpected events at three levels: no, low and high, while continuing to collect participants’ performance (MEI), physiological indicators (HR, HRV), and psychological indicators (STAI).

#### 3.1.1 Participants

Participants in Experiment 2 were recruited using the same criteria and ethical standards described in Experiment 1, except that the participants were selected specifically for their experience operating twin-engine aircraft (DA-42). The sample size calculation indicated that approximately twenty-eight participants were needed. Based on this, twenty-eight participants were recruited from CAFUC. However, three participants were excluded from the data analysis due to imperfect data collection resulting from equipment failure. The remaining twenty-five participants (25 males, 21–30 years, *M*_age_ = 23.36, *SD*_age_ = 2.27) held commercial aviation licenses from CAAC and had logged an average of over 230 h of flight time (231–295 h, *M*_hour_ = 240.67, *SD*_hour_ = 14.02) in simulators and real aircraft.

#### 3.1.2 Instruments

The experimental platform adopts the CNF Simulator FTD D40/D42 models while supporting Flight Management System (FMS) and GPS navigation operations, which can accurately replicate aircraft performance and system feedback (Figure 1C). Furthermore, a POLAR heart (Figure 1B) rate belt is used as the collection device for the heart rate indicator.

#### 3.1.3 Procedures

The experiment required participants to complete three flight tasks of approximately 20 min each. The order in which participants completed the three tasks is counterbalanced to minimize the impact of the learning effect.

The same flight procedure was used for the three conditions of the flight task, all of which were VOR/DME teardrop procedures at one domestic airport. Without unexpected events, the experiment required participants to take off from runway 32 at the airport, climb to a level altitude of 3900 ft at a fixed speed of 90 knots, then increase speed to 100 knots and maintain level flight. After flying to the Final Approach Fix (FAF), it starts descending and executes the final approach procedure until it completes the landing.

The mission, which included a low-urgency unexpected event, required the participant to climb to a level altitude of 3900 ft at a fixed speed of 90 knots, then increase speed to 100 knots and maintain level flight. During level flight, participants encountered an unexpected display system failure 2 nautical miles from the VOR, resulting in pilot trainees losing the heading and attitude information on the primary flight display. At this point, participants were only required to refer to the alternate attitude instrument and magnetic compass to continue the mission with established procedures. After flying 2 nautical miles past the VOR, the display system failure was recovered and participants continued to complete the mission following the procedures. In the high-urgency unexpected event, the failure still occurs during the level flight phase and participants will encounter an engine failure 2 nautical miles from the VOR. Following the engine failure, the pilot trainee was required to complete the appropriate engine protection procedures and attempt to restart the engine. After flying 2 nautical miles past the VOR, the engine failure was recovered and participants continued to follow the procedures to complete the task (Figure 2B).

### 3.2 Data analysis

The data statistics and analysis methods used in Experiment 2 are identical to those used in Experiment 1.

### 3.3 Results

#### 3.3.1 Maneuver Error Index

A repeated-measures ANOVA (Figure 3D) is conducted to analyze the effect of unexpected events on the MEI. Results showed a significant main effect of the type of unexpected events (*F*_(2, 23)_ = 67.15, *p* < 0.001, η_p_^2^ = 0.737). Post hoc multiple comparisons revealed no difference between no unexpected events (*M* = 0.040, *SD* = 0.019) and low-urgency unexpected events (*M* = 0.040, *SD* = 0.012), *p* = 0.970, yet both were significantly lower than the MEI observed under high-urgency unexpected events (*M* = 0.194, *SD* = 0.092), *p* < 0.001. Hypothesis 1a was supported by this result.

Using MATLAB as the computational platform and running the TRAPZ code to generate Supplementary Figure S2, which illustrates the temporal variation in speed, altitude, and heading for a participant during the flight task. The differences between the actual and targeted values are illustrated for a pilot trainee experiencing no, low, and high levels of urgency in response to unexpected events, respectively, in the twin-engine flight scenario. As illustrated by the example, the differences between the actual and targeted speed, altitude, and heading are significantly larger when the pilot trainee encounters a high-urgency unexpected event than a task without unexpected events occurring and a low-urgency unexpected event.

#### 3.3.2 Heart rate

A repeated-measures ANOVA (Figure 3E) is conducted to analyze the effect of unexpected events on the HR. Results showed a significant main effect of the type of unexpected events (*F*_(2, 48)_ = 25.62, *p* < 0.001, η_p_^2^ = 0.516). Post hoc multiple comparisons revealed no difference between no unexpected events (*M* = 84.80, *SD* = 13.97) and low-urgency unexpected events (*M* = 84.62, *SD* = 15.12), *p* = 0.843, yet both were significantly lower than the HR observed under high-urgency unexpected events (*M* = 91.16, *SD* = 15.25), *p* < 0.001. Hypothesis 1b was supported by this result, which provide additional evidence supporting Hypothesis H2 that flight scenario familiarity would moderate the effect of unexpected event urgency on pilot trainees’ physiological responses.

#### 3.3.3 Heart rate variability

As shown in Table 1, repeated-measures ANOVA are conducted on participants’ MEAN NNI, SDNN, CSI and CVI measures respectively.

Results showed significant main effects of the type of unexpected events on MEAN NNI (*F*_(2, 48)_ = 19.09, *p* < 0.001, η_p_^2^ = 0.443), SDNN (*F*_(2, 48)_ = 23.90, *p* < 0.001, η_p_^2^ = 0.499), CSI (*F*_(2, 48)_ = 19.55, *p* < 0.001, η_p_^2^ = 0.449) and CVI (*F*_(2, 48)_ = 4.97, *p* = 0.011, η_p_^2^ = 0.172). For MEAN NNI, *post hoc* multiple comparisons revealed no difference between no unexpected events (*M* = 730.38, *SD* = 122.8) and low-urgency unexpected events (*M* = 734.02, *SD* = 128.47), *p* = 0.919, yet both were significantly higher than the MEAN NNI observed under high-urgency unexpected events (*M* = 684.60, *SD* = 108.47), *p* < 0.001. For SDNN, *post hoc* multiple comparisons revealed no difference between low-urgency unexpected events (*M* = 52.52, *SD* = 23.76) and high-urgency unexpected events (*M* = 51.68, *SD* = 18.89), *p* = 0.765, yet both were significantly lower than the SDNN observed under no unexpected events (*M* = 76.27, *SD* = 35.17), *p* < 0.001. For CSI, *post hoc* multiple comparisons revealed no difference between no unexpected events (*M* = 3.51, *SD* = 1.19) and low-urgency unexpected events (*M* = 3.31, *SD* = 1.00), *p* = 0.408, yet both were significantly lower than the CSI observed under high-urgency unexpected events (*M* = 6.04, *SD* = 3.37), *p* < 0.001. And for CVI, *post hoc* multiple comparisons revealed no difference between low-urgency unexpected events (*M* = 4.32, *SD* = 0.39) and high-urgency unexpected events (*M* = 4.29, *SD* = 0.44), *p* = 0.590, yet both were significantly lower than the CVI observed under no unexpected events (*M* = 4.44, *SD* = 0.40), *p* = 0.001.

#### 3.3.4 Anxiety

A repeated-measures ANOVA (Figure 3F) is conducted to analyze the effect of unexpected events on the state anxiety. Results showed a significant main effect of the type of unexpected events (*F*_(2, 46)_ = 18.20, *p* < 0.001, η_p_^2^ = 0.442). Post hoc multiple comparisons revealed no difference between no unexpected events (*M* = 32.33, *SD* = 7.16) and low-urgency unexpected events (*M* = 33.88, *SD* = 7.86), *p* = 0.885, yet both were significantly lower than the anxiety scores observed under high-urgency unexpected events (*M* = 41.00, *SD* = 12.13), *p* < 0.001. Hypothesis 1c was supported by this result, which provide additional evidence supporting Hypothesis H2 that flight scenario familiarity would moderate the effect of unexpected event urgency on pilot trainees’ psychological responses.

## 4 Discussion

In the present study, two experiments systematically examined how unexpected event urgency and flight scenario familiarity influence pilot trainees performance, physiological states, and psychological responses. Experiment 1 utilized a single-engine scenario (Cessna-172 simulator) involving a low-urgency instrument display failure and a high-urgency aerodynamic stall, while Experiment 2 implemented a twin-engine scenario with an additional no-event baseline condition and modified the high-urgency event to engine failure. The findings indicated that high-urgency unexpected events significantly impaired flight performance, induced higher physiological stress (increased heart rate and sympathetic activation), and elevated psychological anxiety compared to low-urgency and no-event conditions. Notably, scenario familiarity moderated these effects, as pilot trainees demonstrated better overall performance and lower anxiety responses in familiar single-engine scenarios.

### 4.1 Impact of unexpected event urgency on pilot trainees

The significant impact of high-urgency unexpected events observed in this study aligns with previous findings, which indicate that such events typically require immediate action and impose considerable cognitive demands, significantly impairing pilot trainees’ operational performance (Anderson, 2017; Skade et al., 2024; Zenk et al., 2024). High-urgency events necessitate rapid detection, decision-making, and execution of corrective actions, increasing cognitive load and attentive demands. These conditions often lead to cognitive tunneling, where pilot trainees overly focus on threat-related information and neglect other crucial flight parameters, resulting in compromised flight performance (Pooladvand and Hasanzadeh, 2023; Van Der Burg et al., 2025). This aligns with observations from accident analyses, such as Air France Flight 447, where pilot trainees failed to appropriately respond to high-urgency aerodynamic stalls due to cognitive tunneling induced by conflicting airspeed indicators (National Transportation Safety Board, 2010).

Conversely, low-urgency unexpected events, such as instrument display failures, elicited minimal physiological and psychological stress, and had limited negative impacts on performance. Interestingly, in some cases, these low-urgency events improved pilot trainees’ focus and monitoring behaviors, potentially due to moderate arousal facilitating task engagement without overwhelming cognitive resources (Armon et al., 2014; Micula et al., 2022). However, despite the relatively minor immediate risks associated with low-urgency events, the historical case of China Airlines Flight 140 illustrates how inadequate handling of such events can still lead to catastrophic outcomes. This highlights the importance of maintaining adequate vigilance even in less urgent scenarios.

### 4.2 Impact of flight scenario familiarity on pilot trainees

The study also demonstrated the critical role of scenario familiarity in moderating pilot trainees’ responses to unexpected events. Pilot trainees exhibited lower anxiety levels and better flight performance in the single-engine scenarios compared to the twin-engine scenarios. This outcome is consistent with previous findings (Sohn and Doane, 2004), indicating that pilots’ extensive experience and familiarity with certain operational contexts significantly enhance their ability to manage unexpected situations effectively. In the single-engine scenarios, the aerodynamic stall represented a common training event regularly encountered by pilot trainees, thus potentially activating procedural knowledge stored in their long-term memory, allowing efficient and automatic recovery responses. Conversely, the twin-engine scenario introduced greater complexity and operational demands, especially during high-urgency engine failures. Pilot trainees not only needed to address the immediate threat of engine failure but also had to manage additional cognitive tasks such as speed and directional control under asymmetric thrust conditions, substantially increasing their physiological and psychological burden.

In summary, the findings of this research underline the significant impact of unexpected event urgency on pilot trainee performance, physiological responses, and psychological stress. Importantly, scenario familiarity emerged as a crucial factor mitigating these effects, indicating that pilot trainees benefit considerably from prior exposure and operational familiarity with specific flight scenarios. From a practical perspective, these results advocate the necessity of incorporating urgency-based unexpected event training and cross-aircraft proficiency programs into pilot trainee training curricula. Such training would likely enhance pilot trainees’ adaptive responses, reduce cognitive tunneling, and ultimately improve aviation safety and operational effectiveness.

### 4.3 Limitations and future directions

Despite this study provides insights for understanding and handling unexpected events of varying urgency levels, it also has certain limitations. First, one notable limitation is the relatively homogeneous participant pool, consisting primarily of pilot trainees with limited overall flight experience (average flight hours ranging from 230 to 295 h). This constraint may limit the generalizability of the findings to more experienced pilot trainees. Future research could investigate whether similar effects are observed among pilot trainees with varying levels of experience, including more seasoned commercial pilot trainees. Second, the use of flight simulators, while allowing for controlled experimental conditions, does not fully replicate the environmental stressors and physical demands encountered during real flight conditions. Therefore, future studies should consider incorporating real-flight validation where feasible. Finally, physiological data only collected peripheral nervous system indicators such as HR and HRV under unexpected events of varying urgency levels, lacking the collection and analysis of central nervous system signals. Future research could consider integrating technologies like electroencephalography (EEG) and event-related potential (ERP) to analyze the coupling relationship between central and peripheral neural signals from multiple dimensions in the time-frequency domain.

The research findings provide referential ideas and implications for further understanding and exploring unexpected events. For research, this study employed multi-source data to analyze the physiological and psychological characteristics of pilots during unexpected events, providing clues for understanding their cognitive processes when encountering unexpected events of varying urgency levels. The findings indicate that scenario familiarity influences the impact of urgency levels on participants, underscoring the importance of studying unexpected events with different urgency levels across various contexts to develop more universally applicable theories. For airline, these results advocate the necessity of incorporating urgency-based unexpected event training and cross-aircraft proficiency programs into pilot trainee training curricula, in order to enhance the adaptability of pilot trainees and improve aviation safety and operational efficiency.

## 5 Conclusion

In this study, simulated unexpected events of varying urgency levels during flight affect the performance, heart rate, and anxiety levels of pilot trainees, with these effects being moderated by flight scenario familiarity. High-urgency events significantly impaired performance and increased physiological and psychological stress, while low-urgency events had minimal impact and, in some cases, improved focus. Moreover, pilot trainees demonstrated better performance and lower anxiety levels in the more familiar single-engine scenario. These findings suggest that pilot trainees training should incorporate urgency-based scenarios and cross-aircraft training to improve adaptability to unexpected events. Future research should explore these effects in more diverse pilot trainee populations and operational settings, further enhancing aviation safety strategies.

## Data Availability

The datasets presented in this study can be found in online repositories. The names of the repository/repositories and accession number(s) can be found in the article/Supplementary Material.
