# Supplementary material for: Effects of unexpected event urgency and flight scenario familiarity on pilot trainees performance and stress responses
Source: Front Physiol. 2025 Jul 14;16:1599122. doi: 10.3389/fphys.2025.1599122 (PMC12301374; doi:10.3389/fphys.2025.1599122)
Supplement: Supplementary file 1 [file Table1.docx]

Table S1. Term Comparison Table

| Abbreviation | Full Term |
| --- | --- |
| NTSB | National Transportation Safety Board |
| CAFUC | the Civil Aviation Flight University of China |
| CAAC | the Civil Aviation Administration of China |
| IATA | International Air Transport Association |
| PFD | Primary Flight Display |
| MEI | Maneuver Error Index |
| HR | Heart Rate |
| HRV | Heart Rate Variability |
| MEAN NNI | Mean Value of RR Intervals |
| SDNN | Standard Deviation of RR Intervals |
| CVI | Cardiac Vagal Index |
| CSI | Cardiac Sympathetic Index |
| STAI | State-Trait Anxiety Inventory |
| FMS | Flight Management System |
| FAF | Final Approach Fix |
| VOR | Very High Frequency Omnibearing Range |
| DME | Distance Measuring Equipment |
