# Supplementary figures and images for: Effects of unexpected event urgency and flight scenario familiarity on pilot trainees performance and stress responses
Source: Front Physiol. 2025 Jul 14;16:1599122. doi: 10.3389/fphys.2025.1599122 (PMC12301374; doi:10.3389/fphys.2025.1599122)

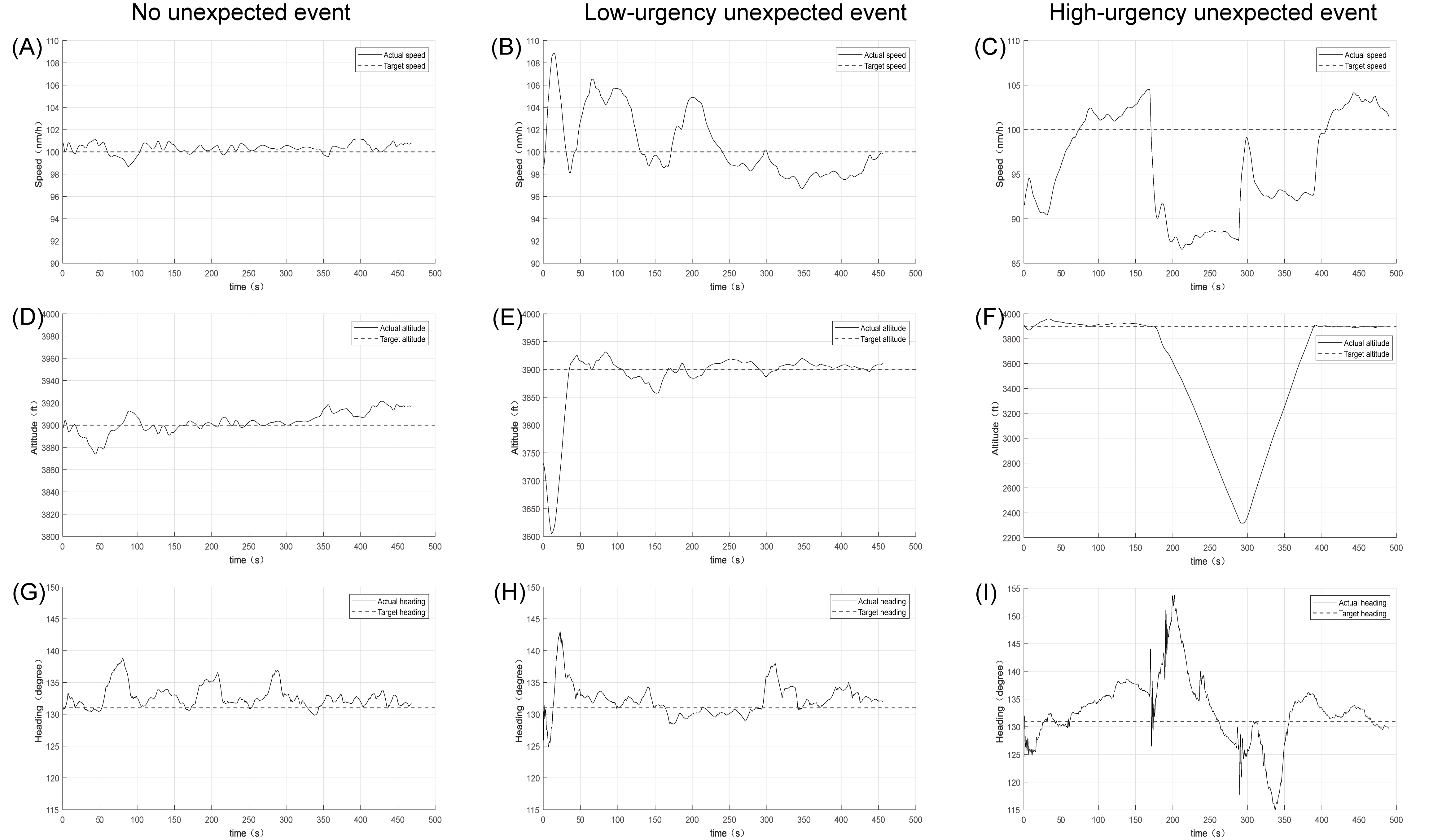

Supplement: Supplementary file 2 [file Image2.tif]

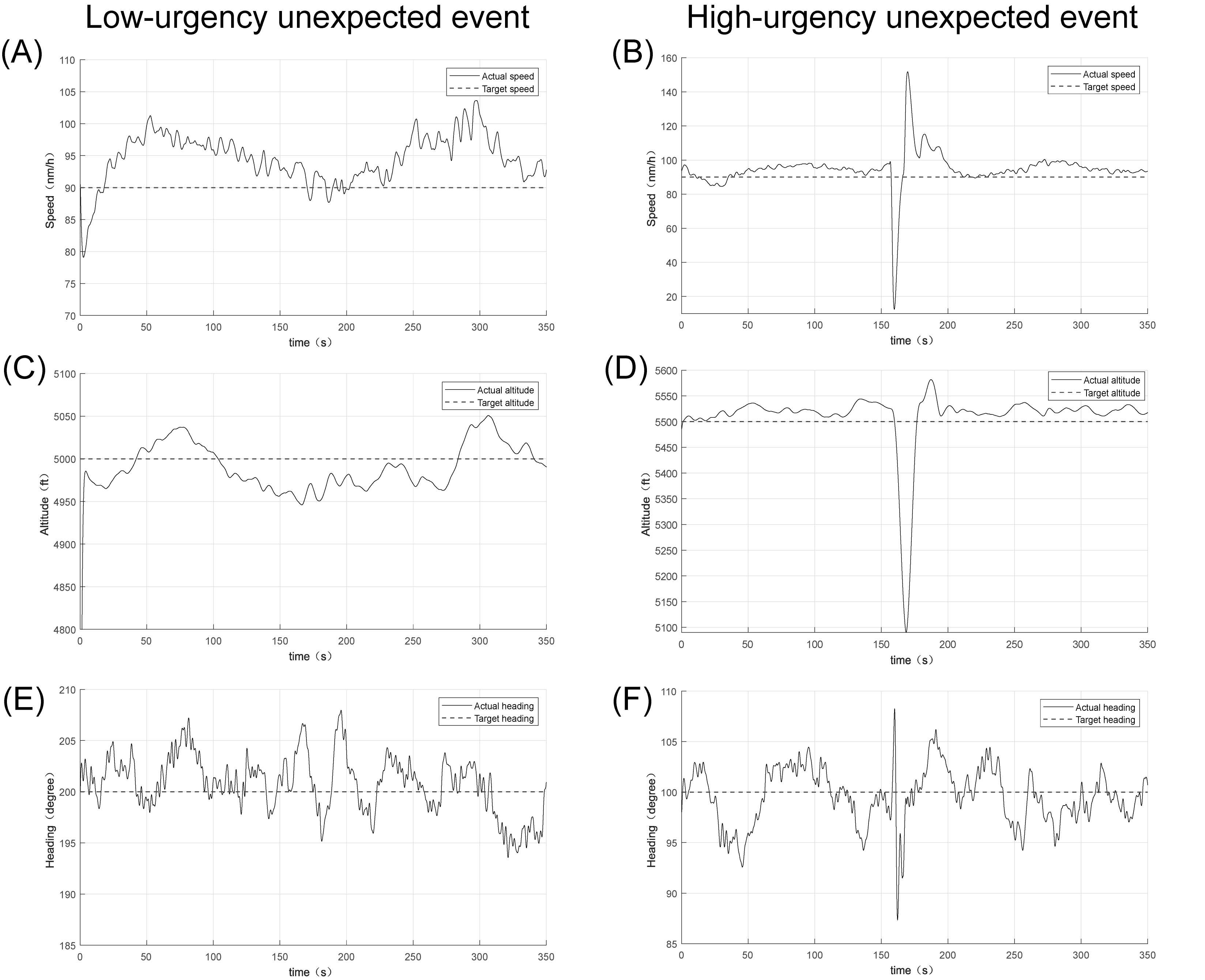

Supplement: Supplementary file 3 [file Image1.tif]
